# Not all who meander are lost: migrating sea lamprey follow river thalwegs to facilitate safe and efficient passage upstream

**DOI:** 10.1242/jeb.249539

**Published:** 2025-02-21

**Authors:** Kandace R. Griffin, Christopher M. Holbrook, Daniel P. Zielinski, Christopher L. Cahill, C. Michael Wagner

**Affiliations:** ^1^ Michigan State University, Department of Fisheries and Wildlife, East Lansing, MI 48824, USA; ^2^ United States Geological Survey, Hammond Bay Biological Station, Millersburg, MI 49759, USA; ^3^ Great Lakes Fishery Commission, Ann Arbor, MI 48105, USA

**Keywords:** Behavior, Estuary, Migration, Predation, Acoustic telemetry, Navigation

## Abstract

Efficient navigation is crucial for the reproductive success of many migratory species, often driven by competing pressures to conserve energy and reduce predation risk. Little is known about how non-homing species achieve this balance. We show that sea lamprey (*Petromyzon marinus*), an ancient extant vertebrate, uses persistent patterns in hydro-geomorphology to quickly and efficiently navigate through complex ecosystems. Hydrodynamic flow models coupled with bathymetric mapping and fine-scale acoustic telemetry revealed movement paths that tracked thalweg scour channels, which are often the deepest and fastest-flowing sections of a river. These paths allow rapid and efficient upstream migration and suggest the existence of a bathymetric highway system. Near-substrate swimming along this path resulted in a median of 5.8% energy savings while also promoting improved safety from nocturnally active predators. We hypothesize sea lampreys use hydrostatic pressure-guided rheotaxis to achieve this navigation. It is likely this tactic relies on sensory information from the animal's primitive lateral line and perhaps the inner ear. Insights from this study can be used to redesign conservation practices to achieve improved control where the animal is invasive and improved fish passage within its native range.

## Teaser

**Highlighted Article:** Migrating sea lamprey appear to use a novel mechanism, hydrostatic pressure-guided rheotaxis, to follow river thalwegs and achieve safe and efficient upstream passage through shallow coastal rivers.

## INTRODUCTION

An animal that undertakes a long-distance reproductive migration must survive the journey and arrive with sufficient time and energy to reproduce. How efficiently individuals complete these tasks has important implications for several ecological and evolutionary processes that determine the success of populations (Dingle, 2014; Fudickar et al., 2021). Thus, it is widely held that natural selection should favor migration strategies that both minimize energy expenditure and reduce predation risk to improve fitness outcomes (Alerstam et al., 2003; Dingle and Drake, 2007; Lennox et al., 2016; Lima and Bednekoff, 1999). This optimization is challenging when the migratory route requires navigation through complex landscapes that exhibit varying energetic costs, uncertainty about the identity and location of predators, and multiple available routes (Bouchet et al., 2015; Crane et al., 2024; Sabal et al., 2021). Examining the environmental features that guide selection of safe and efficient routes can lead to a better understanding of the evolution of sensory-guided movement strategies (notably orientation and navigation). Furthermore, when the population of interest is invasive, elucidation of movement strategies can reveal opportunities to better manage impacts to native species and ecosystems.

With the advent of internal telemetry transmitters, fishes have emerged as accessible experimental systems for *in situ* mechanistic examinations of the orientation tactics used by migrants at fine spatial–temporal scales (Brönmark et al., 2014; Brownscombe et al., 2022; Cooke et al., 2008; Jacoby and Piper, 2023). Many fishes embark on annual spawning migrations that involve traversing estuaries and large rivers to reach distant spawning habitat (Morais and Daverat, 2016). The principal energetic costs are basal metabolism and the cost of swimming against a current (Claridge and Potter, 1975; Kinnison et al., 2001; McElroy et al., 2012). Consequently, there are evolutionary pressures to minimize both the total distance traveled and the rate at which energy is consumed along that route. The latter is hypothesized to involve selecting paths through complex flow fields that allow the fish to avoid swimming against high water velocities (Goodwin et al., 2014; Hintz et al., 2023; Nestler et al., 2012). In rivers, the slowest relative water velocities are most often found in the hydraulically rough flow near the substrate, particularly along riverbanks, where large fishes often migrate [e.g. sockeye salmon (*Oncorhynchus nerka*), Hinch and Rand, 2000; pallid sturgeon (*Scaphirhynchus albus*), McElroy et al., 2012].

Theory also suggests migrating prey should opt to move in alignment with habitat features that promote safety, particularly when the immediacy of danger is difficult to ascertain (Åkesson et al., 2014; Gaynor et al., 2019; Luttbeg et al., 2020; Pfuhl et al., 2011). A common tactic to promote safe passage is to initiate movements during periods of low risk. Often this involves moving at night to avoid visual predators (Chapman et al., 2015; Gwinner, 1996; Reebs, 2002). In fact, most research directed toward understanding safe migration tactics has focused exclusively on movement timing. However, an important consequence of nocturnal movement is increased reliance on non-visual cues for predator detection and orientation, potentially complicating the selection of energetically efficient yet safe routes. This is particularly true for nocturnal species that do not exhibit natal homing and therefore cannot rely on an innate map-and-compass to navigate through the complex morphology of estuaries and river–wetland complexes (Åkesson et al., 2014). However, in rivers, moving nocturnally along shorelines may bring prey fishes into more frequent contact with shoreline predators.

The sea lamprey (*Petromyzon marinus* Linnaeus 1758) is a non-homing (Bergstedt and Seelye, 1995; Waldman et al., 2008) Agnathan fish that is native to the northern Atlantic basin and invasive in the Laurentian Great Lakes. After 1–2 years at sea feeding parasitically on large-bodied fishes, sub-adults embark on a migration to the coastline and through complex estuarine and riverine habitats, relying entirely on stored energy to complete a journey that may be hundreds of kilometers in length and several months in duration (Moser et al., 2015). The sea lamprey is likely to be unaware of its geographic position at the start of the migration as its movements have been those of its host. Orientation toward shore is accomplished by undertaking large circular swims on the substate, ostensibly to sample the local gradient in hydrostatic pressure and align with shallowing water, after which the animal swims near the surface, periodically casting down to the bottom to confirm shallowing depth (Meckley et al., 2017). It selects rivers emitting the odor of conspecific larvae (Sorensen et al., 2005; Vrieze et al., 2010; Wagner et al., 2006, 2009) and transitions to bottom swimming until arriving at the spawning habitat (Holbrook et al., 2015). Throughout this process it remains solitary, relying solely on recent sensory information to complete the migration (McCann et al., 2018).

The potential for considerable weight loss observed during the migration suggests sea lampreys should navigate efficient migration routes to preserve time and energy for spawning (Beamish et al., 1979). How this feat is accomplished is unknown. The animal's apparent use of hydrostatic pressure sensing to orient to water depth during the early migration coupled with the transition to bottom swimming in rivers offers an intriguing possibility. Coastal rivers that drain to the Great Lakes and the Atlantic coast often contain a distinct fluvial thalweg (Albert et al., 2005; Herdendorf, 1990; Larson et al., 2013). In tidally dominated coastal marine systems this channel often merges into ebb and flood tidal scour channels found in estuaries (Dalrymple and Choi, 2007; Leuven et al., 2018), creating a bathymetric ‘highway’ system. Adopting a strategy of occupying the greatest relative depth while migrating upstream should ensure sea lamprey will efficiently migrate through the complex hydrology and morphology of coastal ecosystems without becoming entrained in small embayments or wetland channels whilst benefitting from the energy savings that accompany swimming near the substrate. Furthermore, after entering the shallowing riverine environment, this tactic would allow migrants to occupy the deepest portion of the channel where relative safety from nocturnally active shoreline predators should be improved.

To test the hypothesis that sea lampreys use bathymetry to achieve safe and efficient upstream migration, we examined fine scale movement paths of 56 sea lamprey migrating through a morphologically varied portion of the White River, MI, USA, with areas of distinct thalweg presence and more uniform channel cross-section. We coupled fine scale high-frequency positioning of the fish with a hydrodynamic flow model to test five predictions: (1) sea lamprey persistently swim near the substrate, resulting in (2) a significant energetic cost savings versus swimming higher in the water column; (3) sea lamprey tracks are non-uniformly distributed across the channel exhibiting (4) a preference to move through the deepest portion of the channel; and (5) as a consequence of these patterns, migrants would consistently choose the deeper, higher flow channel at an upstream confluence where two channels of similar width came together.

## MATERIALS AND METHODS

### Study area

**Fig. 1.**
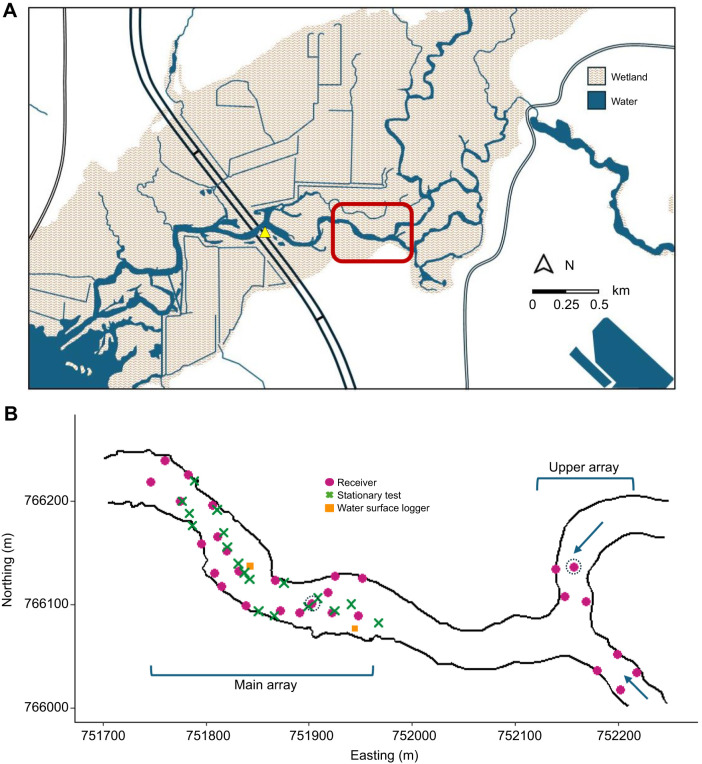
**Location of the study site on the White River, Michigan.** (A) Map of the study area. The study site is indicated by the red rectangle. Tagged sea lampreys were released upstream of US Highway 31 (yellow triangle). (B) Locations of the acoustic receivers with collocated or integrated synchronization tags (pink filled circles), stationary transmitters used in accuracy and precision tests (green crosses) and two water surface level data loggers (orange filled squares). Detections from the two receivers surrounded by dashed lines were not included in analysis (see text for details).

**Fig. 2.**
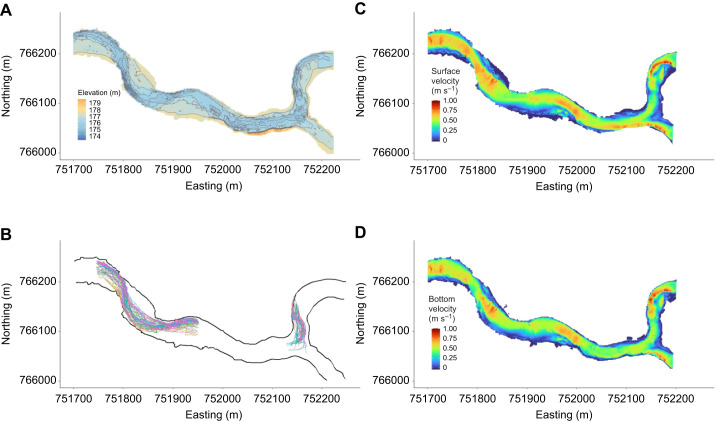
**Sea lamprey tracks and study site bathymetry and water velocity.** (A) Bathymetric map of the study site. (B) Observed fish tracks (*N*=56) through the main and upper arrays after processing and filtering. Each track is colored differently. Map of water velocity (m s^−1^) at standardized height from surface (C) and bottom (D) from the computational fluid dynamics (CFD) model for case 6 (modeled discharge=19.54 m^3^ s^−1^).

Fish tracking took place in the White River near Whitehall, Michigan, USA (43.42°N, 86.32°W), a tributary to Lake Michigan that flows for 134 km through Newaygo, Oceana, and Muskegon Counties with an average discharge of 12.7 m^3^ s^−1^. The study site was situated in the lower watershed where the river cuts through a landscape dominated by wooded wetlands, emergent vegetation beds and open water marshes (Fig. 1A); habitats similar to those of river-dominated estuaries where the sea lamprey migratory strategy evolved. The study consisted of two acoustic telemetry arrays (hereafter called main and upper arrays, Fig. 1B) placed into 0.55 river kilometer (rkm) reach that ranged in depth from 0.1 to 4.23 m and width from 31.7 to 58.5 m, with a sandy substrate throughout. Discharge during the study period ranged from 10.59 to 19.54 m^3^s^−1^. The channel exhibited a mix of asymmetrical cross-sections that included a deep meandering thalweg (sinuosity=1.24) embedded within the straighter channel (sinuosity=1.06) and areas of more uniform cross-sectional depth (Fig. 2A). Shallow depositional areas were present opposite the distinct thalweg that became progressively occupied with submerged aquatic vegetation through late spring and into early summer. The main array was used to address predictions 1–4 through the stretch of asymmetrical and uniform river channel cross-sections. The upper array was placed 0.2 rkm upstream of the main array to investigate lamprey movement decisions at a confluence (prediction 5). Both branches of the confluence rejoin upstream and contain larval odor from larval populations upstream of this point, but each branch exhibited different morphological and hydraulic features with the north branch containing the dominant flow and a distinct thalweg.

### Telemetry array design and testing

Each array was designed by creating squares of equidistant sides constrained by river width to create the overlapping detection range required for fine scale positioning, adjusted to account for line-of-sight challenges due to channel morphology and natural obstacles (e.g. downed trees). Each receiver was attached to a 1.5 m piece of 13 mm diameter steel reinforcing bar using two hose clamps with the hydrophone positioned above the top of the metal rod. During deployment each rod was pressed into the substrate and a 2 m weighted grappling line was attached to aid recovery.

We conducted six diagnostic tests by drifting an acoustic transmitter (hereafter ‘tag’) through the initial main array design near the surface (*n*=3) and near the substrate (*n*=3) to identify potential gaps in detection and subsequent positioning. We identified a 0.4 km^2^ area with low detection efficiency (<47% during one drift near the substrate). We added one supplemental VR2W receiver to the array to increase coverage, improving detection efficiency of the area to 87%. The final main array consisted of acoustic telemetry data-logging receiver models HR2 (18) and VR2W (3) (Innovasea, Nova Scotia, Canada). The upper array included eight VR2W receivers, four in each branch with detection range across the confluence. All receivers operated at 180 kHz and were capable of detecting signals encoded with pulse position modulation (PPM). Additionally, HR2 receivers were capable of detecting tags encoded with binary phase shift keying (HR), which transmitted each code over a much shorter interval (∼1 ms) than a PPM signal (∼1 s). To facilitate time synchronization among receivers, HR2 receivers contained integrated transmitters that emitted an HR-type code every 25–35 s and a PPM-type code every 270–330 s. VR2W receivers did not contain integrated transmitters, so an independent transmitter (Innovasea model V9-2x) was collocated with each VR2W. Final receiver positions were logged with a Trimble Geo XH with positional accuracy of ±10 cm. Upon recovery, one HR2 receiver in the main array had water intrusion which corrupted detection data and the collocated sync tag associated with one VR2W receiver in the upper array failed; detection data from these receivers were not included in positioning. The analysis included data from 17 HR2 and 3 VR2W receivers in the main array, and 7 VR2W receivers in the upper array (Fig. 1B).

Positioning performance of the main array was assessed by comparing the estimated positions to post-processed GPS measured positions (Trimble Geo XH) of collocated sync tags associated with each receiver, stationary tags with known location deployed throughout the study period, and mobile tag tests on 29 June. Stationary reference tests were performed by periodically moving two tags throughout the array during the study period (18 locations total, median test length=26.76 h, Fig. 1B). Mobile tests involved affixing a tag directly below the GPS antenna and drifting through the array (*n*=2, mean test length=781 s). Positional accuracy was measured as the Euclidean distance between array-determined location and the known GPS location for all positions taken from the stationary and mobile tests. Positional precision was estimated for the stationary tests only by calculating the distance between each unique estimated position and the median position for the tag during that test. Notably, tags used in the stationary reference test emitted only PPM code transmissions, whereas mobile tests used a tag transmitting both PPM and HR codes.

### Bathymetric mapping

Bathymetry data were collected with a kayak-mounted side- and bottom-scan sonar unit with internal GPS (Humminbird Helix 7 G3, Johnson Outdoors, AL, USA) on 2 June and 20 July 2021 following procedures described in Kaeser and Litts (2010). We recorded from transects paddled parallel to the shoreline approximately 2 m apart with the sonar frequency set to 455 kHz. Post-collection data processing using SonarTRX software (Leraand Engineering Inc., HI, USA) was performed to apply a slant-range correction and export XYZ (location and depth) files (Kaeser and Litts, 2010). Because water level fluctuated during the study, all depth measurements were standardized to a common vertical datum (NAVD 88) using a continuous water level time series (Fig. 2A). Water level data were recorded with two water surface data loggers (Solinst, Ontario, Canada) every 15 min for the duration of the study (Fig. 1B).

### CFD modeling and validation

The flow field (water velocity and direction) in the White River was modeled using FLOW-3D HYDRO, version 23.1.0.12 (Flow-Science, New Mexico, USA) computational fluid dynamics (CFD) code. FLOW-3D solves the Reynolds-Averages Navier-Stokes equations governing motion for Newtonian incompressible flows using a finite volume method. A two-equation turbulence model, the Renormalization-Group *k*–ε model, was selected because it has wider applicability than the standard *k*–ε model (FLOW-3D User Manual, 2020; Pope, 2000) and better handles low Reynolds number and near-wall flows. A first-order upwind approximation scheme was employed for the momentum advection equations, and an implicit generalized minimum residual method solver was used to determine cell pressures and update the velocity field. The free water surface was tracked using the volume-of-fluid method (Hirt and Nichols, 1981). CFD models were run using a cluster node equipped with a 2.445 GHz AMD EPYC 7763 64-core processor and 32-GB memory.

The bathymetry data was converted to a three-dimensional triangular boundary mesh composed of elements with a side length of 0.5 m using MATLAB (MathWorks, MA, USA), stored as a stereolithography file (STL) and imported into FLOW-3D. Discretization of the geometry and mesh development were completed using fractional area–volume obstacle representation. A single structured mesh was used over the entire domain with rectangular prisms with a uniform cell size of between 2 to 0.5 m in both the horizontal and vertical plane. The upstream boundary condition was specified as a constant discharge, while the downstream boundary condition was specified as a constant water surface elevation. Water surface elevation values were obtained from water level loggers and discharge values were obtained from a combination of acoustic doppler current profiler (ADCP) surveys and a USGS gauge (041222000) located upstream (drainage area ratio of 1.29 between the site and gauge). The CFD model was qualitatively validated using ADCP surveys along four transects conducted on 16 June and 27 July 2021 with river discharge of 9.1 and 10.55 m^3^s^−1^, respectively. For each of the validation discharges, the depth averaged velocity profile along each transect location was compared to ADCP data using a mesh cell size of 2 m, 1 m and 0.5 m (see Fig. S1). The velocity profile matched the ADCP data well for both the 1.0 and 0.5 m mesh cell sizes. A mesh cell size of 0.5 m was used for all subsequent simulations, resulting in a mesh containing ∼1.3 mol l^−1^ elements.

A total of six flow scenarios were developed to simulate flow conditions that fully encompass the range of discharges experienced by sea lampreys in the White River when traveling through the acoustic receiver array (Table 1). To reduce simulation time, each CFD model was first run with a mesh with double resolution for 3000 s of flow time and then 1000 s of flow time were simulated at the finer scale (0.5 m cell size). After 1000 s, the models reached a quasi-steady state in which the total fluid volume within the computational domain reached a plateau and the model outputs were saved. Preliminary post-processing of CFD data was done using TecPlot 360 (TecPlot, Bellevue, Washington). Model output included all cell center coordinates, velocity vector, turbulent kinetic energy (TKE), turbulent intensity (TI), depth averaged velocity magnitude and water surface elevation.

**Table 1.** Summary of water surface elevation (WSEL), total river discharge simulated and number of subjects assigned to each flow scenario

Scenario	WSEL (m)	Discharge range (m^3^ s^−1^)	*n*
1	177.27	10.59	22
2	177.28	11.68	12
3	177.30	12.42	7
4	177.31	14.98	8
5	177.33	16.99	2
6	177.34	19.54	5

### Experimental subjects

Sixty adult sea lampreys were released between 21 May and 11 June 2021 (27 males and 33 females; mean±s.d., total length=50.7±2.9 cm, mass=276.2±38.7 g). Subjects were obtained from barrier traps operated by the US Fish and Wildlife Service during the spawning migration from several rivers in Michigan. Experimental subjects were held in 1385 liter round flow-through tanks that cycled Lake Huron water (100% water exchange every 4 h) with supplemental aeration at Hammond Bay Biological Station (Millersburg, Michigan, USA) until transport to Ludington Biological Station (Ludington, Michigan, USA) where subjects were held prior to tagging and release in a 900 liter recirculating tank (100% water cycle every 1.5 min). Use of sea lampreys and all tagging procedures were approved by the Michigan State University Institutional Animal Use and Care Committee via animal use permit PROTO202100013.

### Tagging and release procedures

Each lamprey was randomly selected and surgically implanted with one of two types of high residence (HR) acoustic transmitters. Each tag emitted two types of coded transmissions with varying pulse delay schedules: a PPM transmission every 13–27 s and a HR transmission every 1.8–2.2 s. Twenty subjects were implanted with V7P tags that transmitted horizontal position and depth via a pressure sensor with each coded transmission (Innovasea model V7P-2x, mass: 1.4 g in air, 0.7 g in water; 7 mm D×19 mm L; power output: 143 dB re. 1 µPa at 1 m). We tested each pressure sensing tag in a pressurized PVC pipe to five levels (0, 3, 6, 9 and 14 PSI) equivalent to 0–10 m. Calibrated slope and intercept values were calculated for each tag. These calibrated values were later used to calculate depth from transmitted sensor values (pressure sensor accuracy: ±0.5 m; resolution: 0.075 m). The remaining 40 animals received V5 tags transmitting two-dimensional positions only (Innovasea model V5-2x, mass: 0.74 g in air, 0.45 g in water; 5.7×5.66 mm D×12.7 mm l; power output: 141 dB re. 1 µPa at 1 m).

Subjects were anesthetized by immersion in 0.9 ml l^−1^ AQUI-S 20 E solution (AQUI-S, New Zealand) for a final concentration of 100 mg l^−1^ eugenol. Lampreys were removed from the anesthetic bath after reaching stage IV of anesthesia, when individuals lost muscle tone but retained gill movement (mean time to stage±s.d., 1200±164 s). After length and weight measurements, individuals were placed in a wet splint foam cradle with fresh water flowing through the mouth and gills maintaining constant gill irrigation. A 10 mm incision was made approximately 10 mm off the ventral midline that ended in line with the anterior insertion of the first dorsal fin to insert the tag in the peritoneal cavity. The incision was closed with two independent interrupted surgeon knots (4-0 Unify sterile PDO monofilament). After each surgical tagging procedure was complete (surgery time, mean±s.d., 185±47 s) animals were monitored in individual postoperative holding tanks until regaining equilibrium, resuming natural swimming and/or attaching to the tank via oral disc (recovery time, mean±s.d., 1262±731 s), and then returned to the post-tagging section of the holding tank. The following day, tagged lampreys were transported to the release site in aerated coolers and acclimated to river water by half volume water exchange until transport tank temperature was within 2°C of river temperature (acclimation time, mean±s.d., 1115±234 s). Lampreys were released into the river 0.9 rkm downstream of the acoustic array between 09:00 and 10:00 h local time (Fig. 1B). Tagging surgeries were performed between 21 May and 10 June, and releases took place between 21 May and 11 June with five or six individuals per release.

### Telemetry data processing

Acoustic telemetry data were processed using Fathom Position software (Innovasea, v.1.8.2) to synchronize time and estimate fish positions. Each receiver's internal clock was corrected to remove clock drift. After time-correction, detection records of collocated tags throughout the array were aligned to time synchronize each receiver to the remaining receivers. Fine-scale positions were calculated within the Fathom Position software by time-difference-of-arrival of acoustic detections arriving at three or more receivers based on hyperbolic principles (MacAulay, 2023; Smith, 2013).

### Position error and filtering

Animal positions were filtered using a three-stage approach (see Supplementary Materials and Methods for more details): (1) only segments of tracks exhibiting upstream movement through the array were included; (2) positions from outside of the array grid were removed; and (3) fish exhibiting sudden implausibly high-speed swimming with an immediate return to the prior track were identified by ground speed and removed (Almeida et al., 2002; Hardisty, 1979; Quintella et al., 2009). Because we were interested in characterizing the migratory route taken during upstream movement and the environmental factors that guide the selection of the route, only positions from upstream movement were included in the analysis. Each estimated position was assigned a measure of error sensitivity, HPE_s_, unitless. Previous studies have used the relationship between HPE_s_ and measured error to remove positions that exceed a threshold (Meckley et al., 2014). In the present study, this relationship did not reveal a meaningful threshold for filtering erroneous positions (see Figs S2 and S3). However, there was clear evidence suggesting positional accuracy degraded considerably as a transmitter moved outside of the array boundary. Thus, we made the decision to exclude positions outside the array boundary. After this exclusion, stationary tests revealed an overall median accuracy (difference in the Euclidean distance from estimated and known position) of 0.41 m, and the two mobile tests had median accuracies of 1.28 m and 1.34 m (Table S1).

After censoring fish positions, visual inspection of fish tracks revealed outliers, e.g. positions on land or a track that exhibited a sudden large lateral movement with an immediate return to the previous trajectory. To identify and remove these errant positions, we applied a filter based on movement speed (per movement speeds reported in Almeida et al., 2002; Hardisty, 1979; Quintella et al., 2009). First, we calculated forward- and backward-looking ground speeds for each step of the track. Positions exceeding 2.5 body lengths s^−1^ in both directions were removed and ground speed was recalculated. At this stage, no errant positions were identified in both directions; however, some positions exceeded 2.5 body lengths s^−1^ in one direction. Next, we calculated gs resulting from the positions before and after this flagged position. If the resulting ground speed was less than 2.5 body lengths s^−1^, the flagged position was removed. After this stage, all forward- and backward-looking ground speeds were considered valid. Tracks were again visually inspected to ensure filtered positions did not compromise the track integrity (Fig. S4).

Prior to analysis, 7765 of 12,218 animal positions were censored from the main array (6610 from stationary fish or fish persistently moving downstream and exiting the array in the downstream direction, 834 from outside the array and 321 from movement speed) and 23 of 393 animal positions were censored from the upper array (22 stopped or downstream movement positions and 1 from movement speed).

### Data analysis

#### Prediction 1: sea lampreys are closely associated with the river bottom

Sea lamprey vertical distribution was described as height in meters from river bottom for the pressure tagged individuals (*n*=18; two fish failed to move upstream during the study period).

#### Prediction 2: the cost of swimming near the bottom is less than swimming higher in the water column

To ascertain the cost savings of swimming near the river bottom, we compared the cost (=work performed) for each discrete step of each movement path when closely associated with the river bottom versus the cost of the same path artificially elevated to near the water surface. To achieve this, each fish position was assigned two elevations to create two identical 2D paths: one at the median height observed from fish with depth sensor tags (0.108 m above the bottom) and another at the same offset from the surface (i.e. 0.108 m below the water surface). For each combination of fish position and elevation, water velocities were extracted from the CFD scenario model that most closely resembled the experienced discharge at time of movement.

The work performed for each lamprey movement track (near-bottom and near-surface) was calculated with equations modified from McElroy et al. (2012), where the environmental conditions encountered over the chosen course influence physical cost (Eqn 1). Notably, this equation assumes drag is the prominent force and other turbulent coherent structures only minorly influence energy requirement which likely does not hold in all conditions. A work value was calculated for each discrete step of the path with the following equation, where ρ is the fluid density (kg m^−3^), *C*_D_ is a dimensionless drag coefficient (Webb, 1975), *S* is the surface area of each fish (m^2^), *U*_r_ is the relative velocity (m s^−1^) and *t* is time (s):
(1)

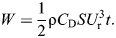


As no drag coefficient has been reported for land-locked sea lamprey, we used the value estimated from the relationship between drag force and water velocity interacting with body surface area for Pacific lamprey (*Entosphenus tridentatus*) which resemble Great Lakes sea lamprey in size and shape, where *C*_D_=0.01 (estimated from fig. 4 in Zobott et al., 2020). The relative velocity, *U*_r_ was calculated over each step, where *d* is the distance (m), *t* is the time (s) and *U*_s_ is the average water velocity magnitude (m s^−1^) between measured locations along a path:
(2)

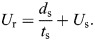


Step lengths had a median value of 2.46 m; therefore, the mean water velocity between adjacent positions on a path is representative of the velocity experienced over that step. We assumed the lamprey body to be oriented to water flow (Binder et al., 2010), rather than the path direction, thus velocity magnitude was used. This might lead to overestimation of *W* in some areas near the surface that exhibit flow reversal (e.g. eddies). Work performed over each step for bottom and surface paths was compared using a one-sided paired Wilcoxon signed-rank test to address the hypothesis that bottom paths had lower cost than surface paths, and we set the significance level for this test at α=0.05.

#### Prediction 3: sea lampreys are non-uniformly distributed across the channel cross-section

To test whether sea lampreys were not uniformly distributed across the river channel, we established transects perpendicular to the river centerline. We then estimated the crossing point for each lamprey path at each transect using linear interpolation from the closest positions upstream and downstream of the transect. We retained transects that were crossed by a minimum of 50 fish (89 transects, mean±s.e.m. distance apart, 2.50±0.07 m). To determine if sea lamprey were nonrandomly distributed across the river channel at each transect, we applied the randomization test described in Holbrook et al. (2015). Each transect was divided into ten equal-width segments, and sea lamprey crossing points were assigned to one segment in each transect. We then compared the difference between observed frequencies of crossing for each segment to expected frequencies drawn from a multinomial distribution of equal probabilities for all ten segments in the transect to calculate a test statistic, χ^2^_obs_. Next, we simulated 10,000 draws from a multinomial distribution representing the 10 intervals of equal probability to compile a distribution of test statistics, χ^2^_null_. To determine a *P*-value, we calculated the proportion of χ^2^_null_ that were more extreme than χ^2^_obs_. The *P*-value was used as a measure of strength of evidence against the null hypothesis of uniform lateral distribution across the river channel, where *P*<0.01, *P*<0.05, *P*<0.10 and *P*>0.10 indicated strong, moderate, weak and no evidence, respectively.

#### Prediction 4: sea lampreys prefer to migrate through the deepest portion of the channel

We used an independent *t*-test to compare the relative water depth usage at each transect for observed and simulated fish to assess relative depth use. Simulated fish positions were randomly drawn from the 10,000 replicates described above at each transect. Relative water depth was calculated by dividing the water depth at each simulated and observed fish position (from the bathymetric mapping) by the maximum water depth of the transect. Consistent significant differences with greater use of deeper segments would indicate a preference for selecting the thalweg.

#### Prediction 5: sea lampreys consistently choose the deeper channel at upstream confluence

Lamprey positions were visually inspected for channel choice and assigned to the north or south channel. To test preference for either channel we performed a one-sample binomial test with the expected proportion of 0.5 for each channel (equal probability of selection).

All analyses were conducted in R version 4.2.3 with the following packages: cmgo, raster, sf (Golly and Turowski, 2017; Pebesma and Bivand, 2023).

## RESULTS

The acoustic telemetry array detected 57 of the 60 tagged individuals at least once. After censoring, positions from 56 animals (31 females, 25 males) were included in the dataset (Fig. 2B). Analysis for predictions 1–4 included 4453 positions in the main array, of which 3108 were 2D and 1345 were 3D positions. The upper array inspection (prediction 5) included 370 positions. Positions per individual ranged from 34 to 189 (median 81).

**Fig. 3.**
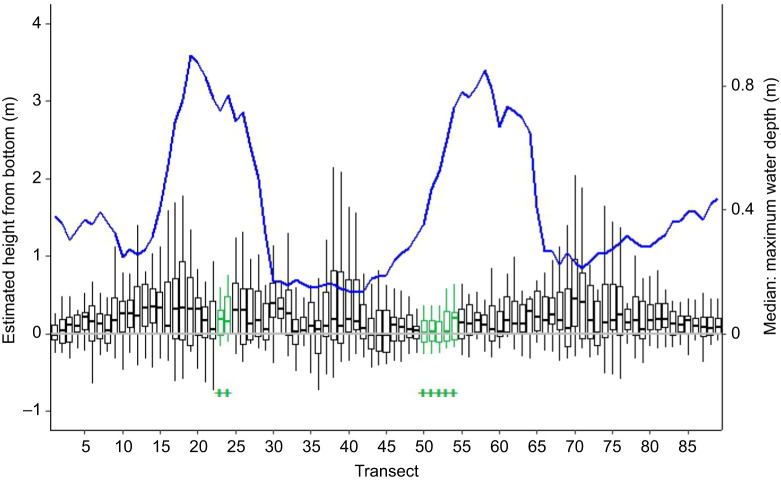
**The vertical distribution of pressure-sensory tagged sea lampreys (*n*=18) moving through the main array at 89 transects.** The ratio of median to maximum water depth at each transect is represented by the solid blue line. High values of this ratio are associated with areas of the stream with a more uniform cross-section. Vertical distributions are described as estimated height from bottom (m). Boxes indicate interquartile range (IQR), solid line between corresponds to median height, and whiskers represent the highest and lowest value within 1.5×IQR. Solid horizontal grey line indicates the river bottom. Distributions in green and indicated with+refer to transects where lampreys did not reveal a preference for the deepest portion of the transect, where observed fish (*N*=56) relative depth usage did not differ significantly from simulated fish relative depth.

**Fig. 4.**
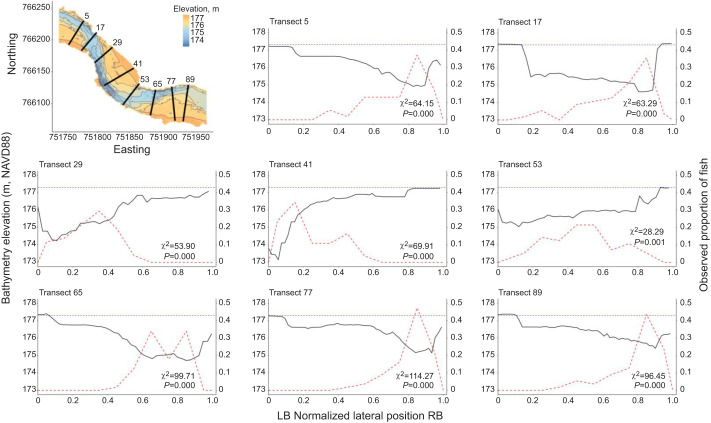
**Normalized lateral distribution of fish and water depth at eight representative transects.** Subplots for the individual transects show the depth profile (solid black line) relative to the average water surface elevation (blue dotted line) and the observed proportion of fish in each of 10 bins representing 10% of the normalized width (red dashed line). χ^2^ and *P*-values indicate significant difference from a uniform fish distribution for the transect. LB, left bank; RB, right bank.

**Fig. 5.**
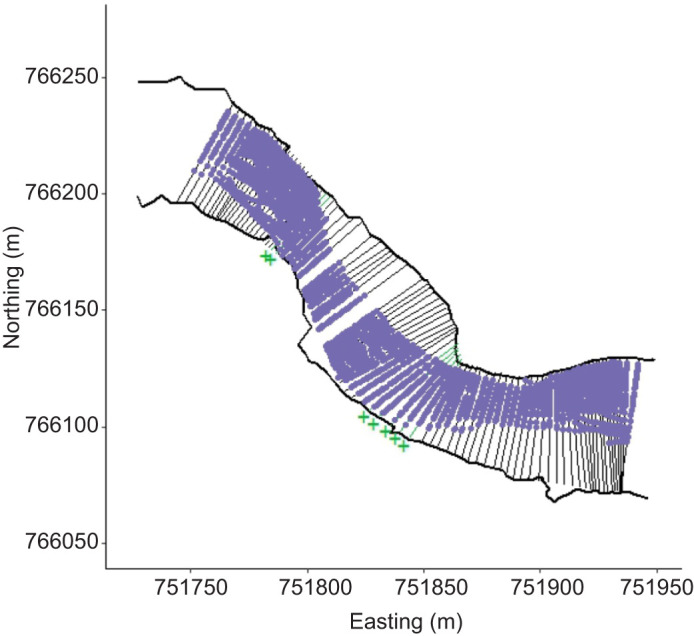
**Observed fish positions (*N*=50–56) on each of the 89 transects.** Transects marked with green+indicate non-significant difference between observed and simulated fish relative depth usage.

Three-dimension positions revealed close association with the river bottom regardless of lateral position (prediction 1). At the 89 transects crossed by at least 50 fish, the median distance from the bottom for the 18 animals transmitting 3D positions ranged from −0.02 to 0.49 m (mean=0.25 m) (Fig. 3). Consistent with prediction 3, sea lampreys were non-uniformly distributed laterally across the channel at each of the 89 transects (χ^2^ range=27.93–126.14, all *P*<0.001, indicating strong evidence for rejecting the null hypothesis of a uniform use of the channel cross-section, Fig. 3). Lamprey positions on the transects were consistent with a preference for moving through the deeper portions of the channel (prediction 4, Figs 2 and 4). For 82 of 89 transects, *t*-tests revealed a preference for movement through the deeper portion of the river (*t*=2.07–9.10, all *P*<0.04). Lamprey positions were in water 23% deeper than predictions from modeled fish paths (observed mean (±1 s) relative depth, 0.68±0.004 m; predicted mean relative depth with uniform distribution, 0.45±0.004 m). Twenty-six individuals consistently chose the deepest quarter of the river, with median relative water depths greater than 0.75 m (median relative water depth per individual range=0.38–0.90 m). Lampreys did not exhibit a preference for moving through the deepest part of the channel at 7 transects (transects 23, 24, 50–54 labeled with green crosses in Figs 3 and Fig. 5). These transects aligned with portions of the river channel exhibiting a more uniform cross-section (i.e. the ratios of median depth to maximum depth in Fig. 3 were approaching 1). Consistent with prediction 2, swimming at the surface (median cost per step=52.43 J; mean cost per m=22.64 J m^−1^) was on average 5.8% more costly than swimming near the bottom (median cost per step=49.76 J; mean cost per m=21.67 J m^−1^) of the river (*Z*=−9.48; d.f.=4396, *P*<0.0001; *r*=0.14). All 56 individuals chose the deeper north channel that also had greater discharge (prediction 5, *z*=7.35, *P*<0.0001; Fig. 2C).

## DISCUSSION

This study demonstrates that sea lampreys on the spawning migration moved in close association with the river bottom achieving a significant energetic savings relative to moving higher in the water column. Additionally, we observed a distinct pattern of thalweg tracking in shallow river systems, with migrants adjusting their lateral position to remain in the deepest areas. When encountering a confluence, all lampreys chose the deeper channel with greater flow despite both channels exhibiting areas of relatively high water velocity (Fig. 2C,D). These insights have important implications for both the management and conservation of sea lamprey populations, providing opportunities to improve trapping efficiency and design more effective fish passage systems.

Fishes that undertake long or costly migrations are more likely to engage in energy saving swim tactics than those that move short distances (Bernatchez and Dodson, 1987). Swimming in close association with the river substrate is consistent with minimizing the rate of energy expenditure by swimming against slower velocity water. In the White River, sea lamprey maintained a median height above bottom of 0.108 m, comparable but slightly lower than the 0.16 m median height observed in the deeper Mississagi River (Ontario, Canada; Holbrook et al., 2015). This resulted in a mean energy savings of 5.8% relative to the modeled cost of swimming identical paths higher in the water column. Notably, our study took place during an unusually dry spring. Sea lamprey often time upstream movement to coincide with freshets (Almeida et al., 2002; Morman et al., 1980). During floods the relative cost savings associated with bottom swimming should be greater. The practical effect of this behavior is to allow the animal to maintain a desired ground speed at a slower tail beat frequency, preventing exhaustion over long periods of sustained swimming (Beamish, 1974; Hoover and Murphy, 2018). We anticipate that efficient navigation upstream may require lampreys to move out of boundary layer flow nearest the substrate (lower mean velocities but higher relative turbulence) and into the lower layers of the bulk flow (higher mean velocity with lower relative turbulence) to maintain rheotactic alignment with the bulk flow. The distance above channel bottom lamprey must reach to encounter bulk flow is likely to be variable owing to changes in channel topography, substrate material and discharge, which influence the boundary layer thickness. This tactic should lead to preservation of energy for predator avoidance, mate search and assessment, gamete development, nest building and spawning activity (Alerstam et al., 2003; Johnson et al., 2015; Lennox et al., 2016).

Swimming in close association with the bottom should also allow sea lampreys to determine the depth of the water column by sensing hydrostatic pressure (Burt de Perera et al., 2005; Davis et al., 2021). There is evidence that sea lampreys use hydrostatic pressure to orient and navigate towards coastlines early in the spawning migration (Meckley et al., 2017). The sea lamprey's ability to sense hydrostatic pressure may involve the vestibular hairs of the inner ear (Maklad et al., 2014) and/or superior neuromasts on the epidermis. Other fishes have been shown to detect hydrostatic pressure using the lateral line, inner ear and swim bladder (Bleckmann and Zelick, 2009; Fraser et al., 2008). Given that sea lamprey lack a swim bladder and have a primitive lateral line and that the sea lamprey inner ear exhibits structural similarity with other pressure-sensitive fishes, the inner ear may house the ability to detect changing water depth, although the lateral line cannot be excluded (Fraser et al., 2008; Hammond and Whitfield, 2006; Khorevin, 2008; Maklad et al., 2014).

When swimming near the bottom, the perception of reducing hydrostatic pressure may indicate the ecosystem is shallowing or the animal has moved close to the riverbank. Either circumstance should increase the risk of encountering nocturnal shoreline and submerged predators that feed on migrating lampreys (Boulêtreau et al., 2020; Cochran, 2009; Harvey and White, 2017). In a study of European river lampreys (*Lampetra fluviatilis*) migrating through a shallow river reach of uniform cross-section, migrants were observed avoiding river edges (Kerr et al., 2023). We hypothesize that the sea lamprey has evolved to avoid areas deemed too shallow to be safe and to avoid near-surface swimming. These tactics would also assist in avoiding entrainment into fringing wetlands or coves when moving through estuaries and river–wetland complexes. Sea lampreys are also known to increase ground speed when exposed to a danger cue in small shallow rivers (Luhring et al., 2016). Whether they show similar acceleration when moving through a shallow reach of a river or during low flows is currently unknown. Additionally, there may be a water depth that labels a location as sufficiently safe. In these ‘safe enough’ portions of the river (i.e. sufficiently deep), a tactic of movement through areas of highest cost savings such as near the bottom and closer to the riverbanks where water velocity is lower than the main channel may be favored, as observed by Holbrook et al. (2015).

We hypothesize that sea lampreys use a novel behavior to navigate through shallow river and estuarine systems by principally aligning opposed to water direction and using hydrostatic pressure to make lateral course corrections to stay in the deepest part of the channel. In the relatively shallow White River, migrating sea lamprey movement paths exhibited a non-uniform distribution across the channel with a distinct preference for moving through the deepest areas. Wherever a distinct thalweg was present, movement paths converged in alignment with the thalweg. When the channel transitioned to a more uniform cross-section, movement paths diverged and spread across the channel while continuing to avoid the shallow margins, reconverging when the thalweg reemerged. This pattern of thalweg tracking is consistent with navigation in response to changing hydrostatic pressure via pressure guided rheotaxis. Lampreys have a lateral line system comprising superficial neuromasts capable of detecting changes in water velocity and direction that allows migrants to orient upstream (Gelman et al., 2007, 2008; Johnson et al., 2012; Katori et al., 1994; Yamada, 1973). If the direction of movement becomes misaligned with the thalweg, we anticipate the animal detects a lessening hydrostatic pressure and can execute a contralateral movement to remain in the deeper parts of the channel. This tactic would naturally result in the animals becoming more dispersed in uniform cross-section reaches of the stream as the animal would not perceive a change in hydrostatic pressure with lateral drift and would continue to orient into the bulk flow. This pattern was observed by Kerr et al. (2023) when observing river lamprey migrating through a river with a uniform cross-section.

Migration through estuaries, drowned river mouths and river–wetland complexes involves selecting movement pathways through complex channel bedforms and hydraulic fields that shift over time and space (Nestler et al., 2012). Because scour channels are persistent morphological features of estuarine and river bottoms that predictably lead to the upper watershed, a migration strategy that includes movement through these features should reduce the time needed to arrive at the spawning habitat. For example, in tidally dominated estuaries, it is typical to find single or dual channels cut into the bed by ebb and flood tidal scour that eventually link into the fluvial thalweg (Dalrymple and Choi, 2007). This hydro-geomorphological pattern is common to many estuaries and rivers that currently or historically recruited large migrations of sea lamprey from the Atlantic Ocean [e.g. Ouse–Humber estuary (Karunarathna et al., 2008; Silva et al., 2017), Scheldt River estuary (Leuven et al., 2018)]. Because the Great Lakes do not experience tides, only the fluvial thalweg is present in shallow coastal river–wetland complexes, often extending to the river mouth and slightly offshore (Herdendorf, 1990; Larson et al., 2013). Use of pressure-guided rheotaxis would allow migrants to navigate upstream within the scour channels and thereby swim a relatively short path through the riverscape by avoiding diversion into minor side channels associated with wetlands, marshes and tributaries. In effect, following a ‘bathymetric highway’ system. Moreover, because the relationship between channel shape and hydrodynamic forces that structure estuarine and riverine bedforms are persistent over geological time, allegiance to relative depth and flow values would allow successive generations to successfully navigate as channels shift position in response to variation in river discharge, sediment loads, and tides. How then do they choose in places where multiple erosional channels diverge? Superimposed on the hydro-geomorphological ‘roadmap’ is larval odor, a conspecific cue that labels habitat as suitable for reproduction and guides migrants into spawning streams (Sorensen et al., 2005). The odor is carried downstream and migrating sea lampreys avoid swimming through waters that do not contain the odor, preventing entry into tributaries that lack larval populations (Wagner et al., 2006, 2009). Similar reinforcement may occur when moving through estuaries and freshwater river-wetland complexes; however, given the extensive mixing that occurs in estuarine systems, hydrogeomorphology may provide a more consistent and distinct orientation cue.

Upstream movement through the thalweg should also result in increased safety. Remaining on the bottom in the deepest portion of the river likely decreases detection by nocturnal shoreline predators, particularly when the thalweg meanders into close proximity to the riverbank (Boulêtreau et al., 2020; Imre et al., 2014). In shallow rivers, remaining in the deepest portion reduces the chance of breaking the water surface, thus minimizing the risk of detection by aerial or surface-dwelling predators. Sea lampreys exhibit a dark dorsal surface and counter shading that should also reduce predator detection by visually hunting predators. Although thalweg tracking optimizes the physical position within the river to reduce encounters with predators, countershading ensures that any potential sightings are less likely to result in detection or predation. Moving through areas with higher water velocity near the surface, which are less desirable hunting grounds for many predators, further aids in avoiding grappling and ambush tactics commonly employed by predatory species (Hart and Finelli, 1999; Peckarsky et al., 1990). Additionally, by reducing the total migration time (increased navigational efficiency) thalweg tracking also reduces the time exposed to predators (Pinti et al., 2020).

We expect the navigational tactics observed in the White River to arise in similar systems. That is, relatively shallow rivers (≤5 m deep) embedded in wetland complexes or estuaries that contain distinct scour channels in the bed. We are aware of two other studies of fine-scale lamprey navigation in rivers during the spawning migration. Kerr et al. (2023) evaluated movement paths of migrating adult European river lamprey in a shallow river with uniform cross-section. They observed patterns similar to those in the portion of our study reach that also had a uniform cross-section: upstream oriented paths widely distributed across the channel with avoidance of the shallow margins. They (and we) conclude that eschewing movement through shallow river margins would result in avoiding slow-moving waters where rheotaxis would be difficult to maintain. We further posit that avoidance of shallow areas will help migrants avoid entering tributary channels leading into surrounding wetlands. In our study, sea lampreys approached a confluence of roughly equal-width channels from a uniform cross-section reach, with all individuals selecting the deeper route associated with the mainstem of the river. The alternative route led to a shallow and heavily vegetated channel that eventually reconnected with the main river. Consequently, the choice was not affected by larval odor, as the populations of larvae were upstream of where the two channels reconnected. This selection may have been reinforced by the higher discharge in the mainstem.

In the other fine-scale lamprey spawning migration study, Holbrook et al. (2015) investigated sea lamprey movement paths in the Mississagi River, a tributary to Lake Huron, in a 1.22 km reach that was 3–6 m deep in the main channel descending to >15 m in a river bend. Here, sea lampreys moved along the bottom and in the straight sections of the study area were more likely to move between the river's edges and the thalweg. The findings of the three studies can be reconciled through the lens of optimization of the two dominant and competing selective pressures: minimizing energy expenditure while also maximizing avoidance of predators. In most northern temperate zone rivers occupied by sea lampreys, the least expensive path will coincide with the riskiest path, movement along the river margin, when predation pressure is coming from nocturnal shoreline mammals (Cochran, 2009; McElroy et al., 2012). If the animal relies on hydrostatic pressure to avoid shorelines, it is functionally avoiding shallow areas regardless of their location. We can imagine this as a lens of water at the surface with a thickness equal to the depth the animal deems safe. Near the shoreline this lens of unsafe water would intersect with the substrate rendering the full water column unsafe. Likewise, the zone of energetic savings is associated with swimming near the substrate, with the greatest energetic savings near the riverbank where flow is slowest and the least savings under the fastest flow, typically in the thalweg. In shallow systems with a distinct thalweg, much or all the river margins may be unacceptably shallow (i.e. unsafe), causing animals to move through the thalweg. Where the channel becomes more uniform, all but the shallow margins represent the deepest area, leading to dispersion of movement paths consistent with our study and that of Kerr et al. (2023). In a deeper river with steeper banks, as in Holbrook et al. (2015), migrants could move closer to the riverbanks to benefit from greater energetic savings while remaining sufficiently deep to promote safety. We would expect a pattern similar to that observed in deeper rivers to occur in shallow rivers during spring high flow events, a time of high migration (Moser et al., 2015). During spates, water depths would increase, as would water velocities near the thalweg. However, when the river passes through wetland-dominated landscapes, as in our study site, thalweg tracking may persist during floods. The wetland surface surrounding the White River site was typically 20–30 cm above the water surface at base flow. Floods greatly increased flow, but not surface elevation, as the water spilled onto the wetland surface.

The ability to anticipate where and when an animal will move presents opportunities to improve conservation and management actions. In the Great Lakes basin, there is a considerable desire to develop methods for invasive sea lamprey control that do not rely on the application of pesticides (Burkett et al., 2021; Lewandoski et al., 2021; Siefkes et al., 2021), including the development of effective means to fish migrating sea lampreys prior to spawning (McLaughlin et al., 2007; Miehls et al., 2020). Current and historical approaches to fishing sea lamprey for control invariably focus on unbaited traps placed in association with dams (Miehls et al., 2021). Many of these traps experience low and variable success because of low and inconsistent encounter rates with migrants (Bravener and McLaughlin, 2013), especially when trap entrances are placed well above the substrate amidst high velocity turbulent flow (Rous et al., 2017). Our findings suggest evaluation of channel morphology and hydrology lampreys experience as they approach the barrier and the fishing device location could be useful in diagnosing why certain barrier-integrated traps fail (e.g. their position is misaligned with preferred movement paths) and might also be modified to improve trap encounter rates. Moreover, the patterns we observed suggest fishing sea lamprey in open river channels away from dams is viable. Fishing practices should involve placement of fishing devices in meandering thalwegs where they run adjacent to the riverbank, targeting reaches that flow through flat landscapes that experience relatively small changes in depth during floods. Within their native ranges, there is also a strong desire to create opportunities to pass several migrating lamprey species around dams (Moser et al., 2021). Because fish passage devices are placed in association with dams, typically at the riverbank, an examination of the hydro-geomorphological features that guide movement downstream of the dam may also help to diagnose unsatisfactory performance. For example, a study conducted in the River Mondego (Portugal) revealed anadromous sea lamprey were more likely to attempt passage of a dam during low flow, focusing their attempts near the center of channel (deeper, faster flow), well away from the fish passage device (Pereira et al., 2017). Because dams are often sited in places with uniform and geologically stable cross-sections, improving success of failing fish passage devices may involve reengineering the approach via dredging or other modifications to the channel geometry. Whether trapping for control or attempting fish passage, our findings reinforce those of other workers who suggest device entrances should be placed near the river bottom (Holbrook et al., 2015; Rous et al., 2017).

### Conclusion

Scientists have long been fascinated by the movement strategies animals use to complete long-distance reproductive migrations. These efforts have focused on homing animals that utilize geomagnetic information and map-and-compass navigation. Notably, many migrating animals neither home nor use this mechanism (Chapman et al., 2014). Our study has revealed a novel navigational tactic utilized by non-homing sea lampreys that involves optimization of competing selective pressures to minimize energy expenditure predation risk by relying on physical features of river morphology and hydrology that persist over geological time. Remarkably, this tactic likely requires inputs from only two sensory systems: the lateral line for detecting water velocity and direction, and an unknown mechanism for sensing hydrostatic pressure changes (possibly superior neuromasts and/or the inner ear). Insights from this and similar studies offer significant opportunities to improve the performance of conservation actions related to invasive species control and population enhancement. We suggest the coupling of fine-scale, high-frequency recording of animal positions with carefully constructed mechanistic hypotheses of movement strategies will prove a powerful approach to designing, reconfiguring and operating infrastructure (fishing devices and dams) to achieve improved outcomes.

## Supplementary Material

10.1242/jexbio.249539_sup1 Supplementary information
