# Supplementary material for: Not all who meander are lost: migrating sea lamprey follow river thalwegs to facilitate safe and efficient passage upstream
Source: J Exp Biol. 2025 Feb 21;228(4):JEB249539. doi: 10.1242/jeb.249539 (PMC11883278; doi:10.1242/jeb.249539)
Supplement: Supplementary information [file jexbio-228-249539-s1.pdf]

Validating CFD with ADCP surveys

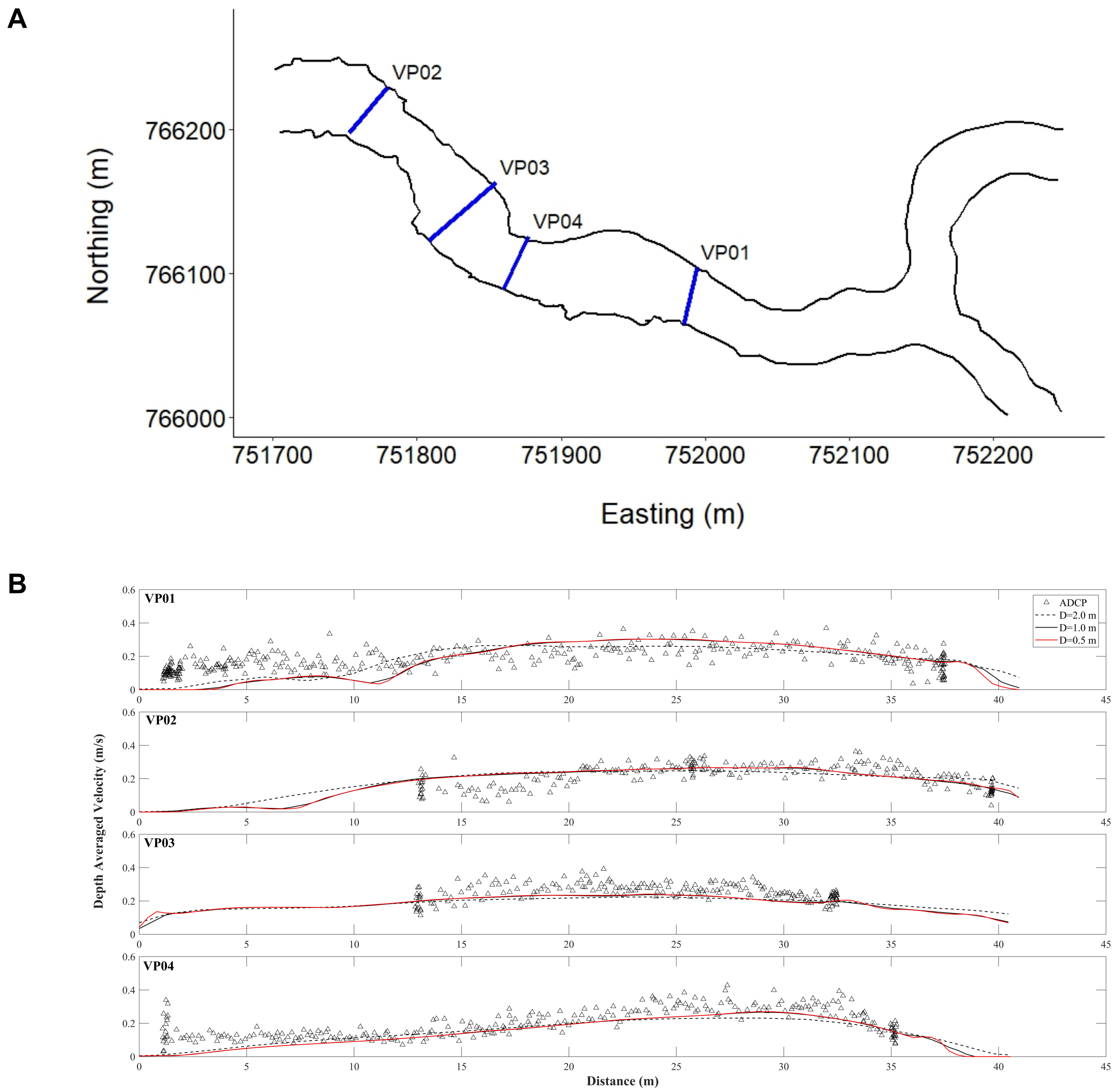

Fig. S1. Comparison of depth averaged velocity measurements at four ADCP survey transects taken along the White River on 16 June 2021. (A) Map indicating acoustic doppler current profiler (ADCP) survey transects with solid blue lines. (B) The ADCP velocity measurements are indicated by black triangles and CFD values are indicated by the red, black, and black-dashed lines.

Journal of Experimental Biology • Supplementary information

Supplementary Materials and Methods

Fish position error estimation and data filtering

Each estimated fish position was assigned an estimate of horizontal position error sensitivity (HPE_s, unitless) and transmitters with known locations (collocated with receivers) were also assigned measurements of horizontal position error (HPE_m, m) where measured GPS location was known. We calculated the relationship between Fathom-provided HPE_m and HPE_s for the collocated receiver positions to apply to fish position HPE_s values to estimate absolute position error (HPE_m^*, m). To limit the influence of positions with high uncertainty on the calculated relationship, we excluded positions with HPE_s values in the 95th percentile (Smith, 2013). Although this exclusion resulted in 104,536 sync positions, the range of HPE_s values was limited to 0.47 – 4.74. We calculated the twice distance root mean squared (2DRMS) as twice the square root of the combined variance in the X (easting) and Y (northing) direction for each 1-m bin averaged over all receivers (Fig. S2). Next, to estimate HPE_m^* we applied the slope and intercept of the linear regression of HPE_s and 2DRMS to HPE_s values (Meckley et al., 2014; Smith, 2013).

To validate this approach, we calculated HPE_m^* using the same slope and intercept for the 11,047 stationary and 379 mobile tests positions and compared that value to measured positional error (i.e., the Euclidean distance between known GPS location and estimated position). This comparison did not result in the expected 1:1 relationship between HPE_m^* and measured error (regression equation: HPE_m^* = 0.821 + 7.14 * measured error; R^2 = 0.08). Instead, high HPE_m^* values were assigned to positions with low measured positional error therefore overestimating the positional error. For example, applying the HPE_m^* filter of 5 m to the mobile test positions identified 49% of positions (n = 185) as erroneous, but the median accuracy of those positions was 1.82 m (mean = 5.49 m) (Fig. S3).

HPE_m^* did not offer a useful threshold for filtering erroneous positions within the array. However, there was clear evidence from both known positional error and HPE_m values suggesting positional accuracy degraded considerably as a transmitter moved outside of the array boundary. After censoring stationary and mobile tests to exclude positions outside of the upstream-most and downstream-most receivers, the 17 resulting stationary tests (Table S1) revealed an overall mean accuracy (± 1 SE) of 1.03 ± 0.04 m (median 0.41 m; mean range of each test 0.39 – 3.39 m), and the 2 tag drags had a mean accuracy (± 1 SE) of 1.70 ± 0.15 m (median = 1.28 m) and 2.37 ± 0.41 m (median = 1.34 m).

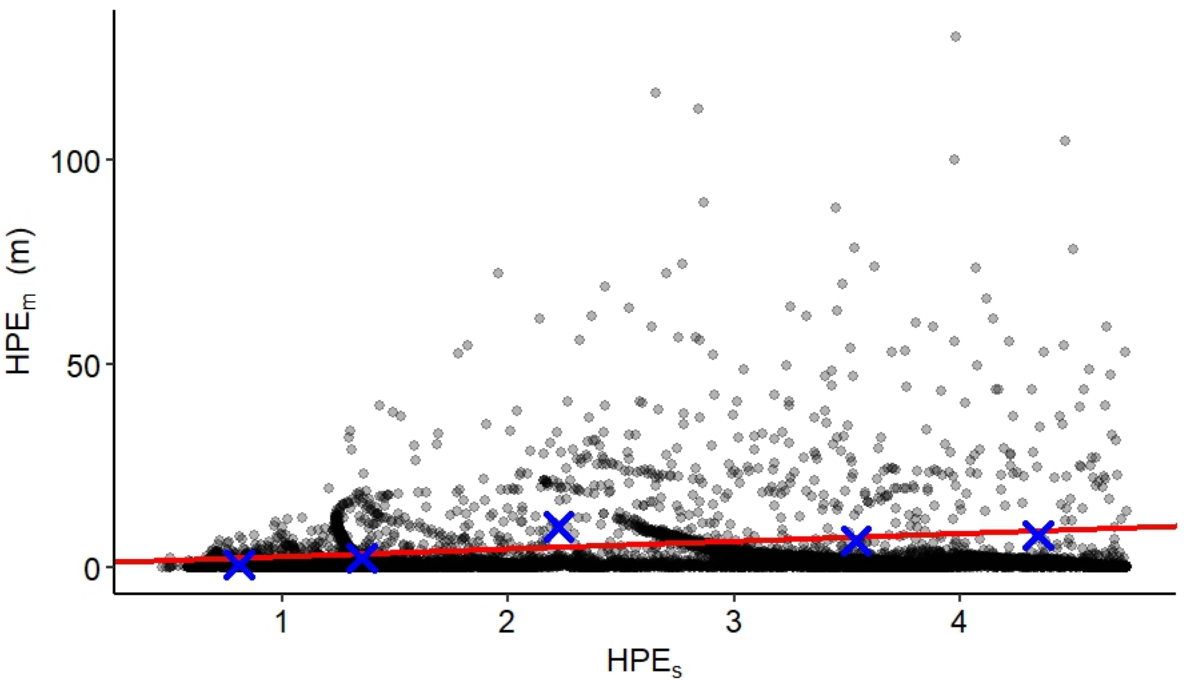

Fig. S2. Fathom-provided horizontal position error (HPE_m) versus horizontal position error sensitivity (HPE_s) values for each estimated collocated sync tag position. The blue x represents twice the distance root mean square error of X and Y components of error within an HPE_s bin of one; 2DRMS is represented by the solid red line running between these points.

Journal of Experimental Biology • Supplementary information

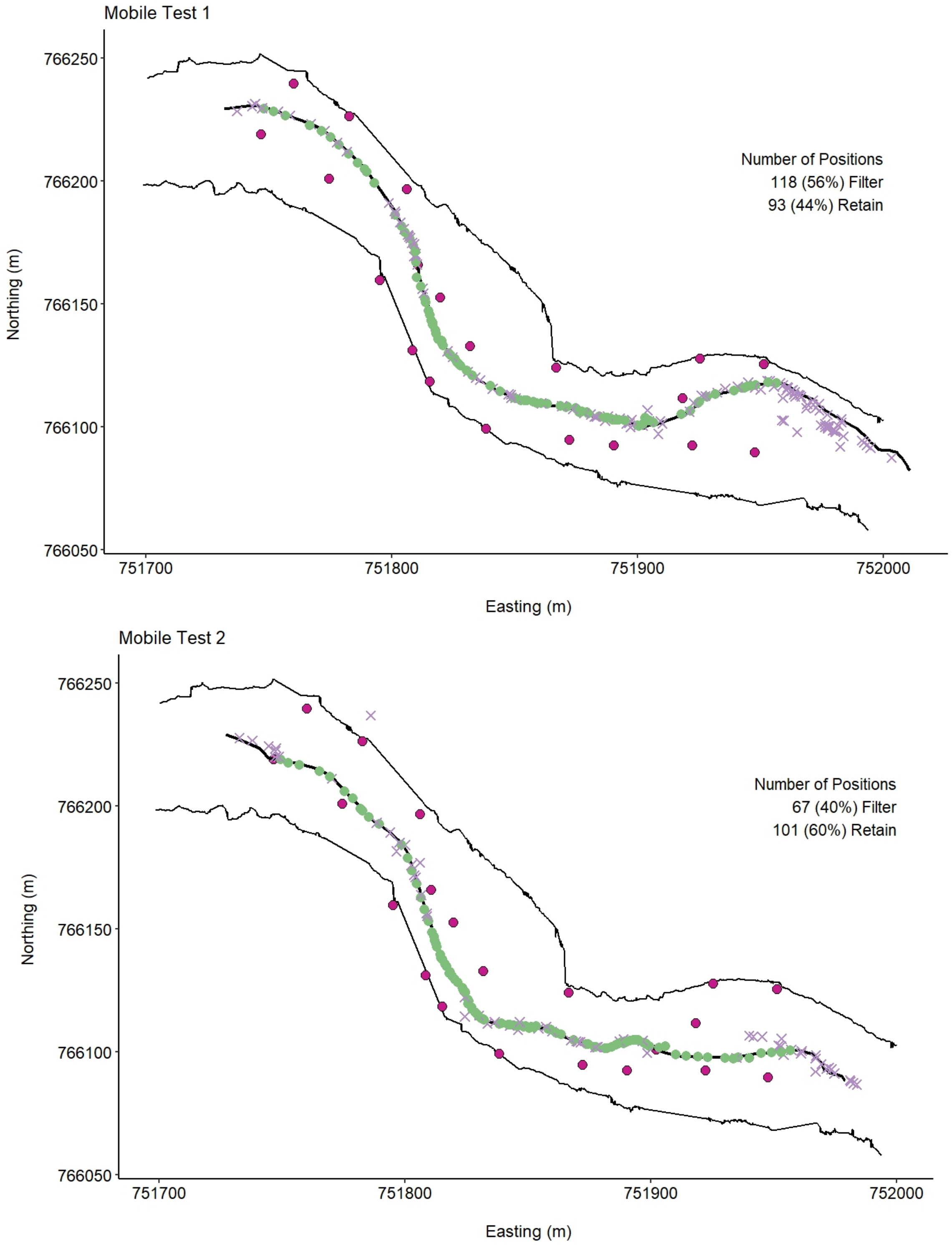

Fig. S3. Estimated positions and known GPS track from mobile tests. Positions retained (green circle) and filtered (purple crosses) from mobile tests when applying HPE_m^* filter of 5 m. Acoustic receivers are indicated by pink circles. The solid black line indicates the known GPS track.

Journal of Experimental Biology • Supplementary information

Table S1. Accuracy and precision of stationary tests (*N* = 17) after censoring positions outside the receiver array.

Test	n Positions	Accuracy (m)				Precision (m)				Test Length (hr)	Fix Rate
		Mean	SD	SE	Median	Mean	SD	SE	Median		
1	1280	0.49	2.69	0.08	0.18	0.40	2.70	0.08	0.07	120.59	88.90
2	989	1.07	6.06	0.19	0.34	0.87	6.11	0.19	0.11	90.75	91.27
3	419	0.97	4.66	0.23	0.30	0.77	4.69	0.23	0.07	39.17	89.59
4	294	0.71	4.15	0.24	0.20	0.61	4.16	0.24	0.09	27.72	88.83
5	244	0.39	0.99	0.06	0.25	0.25	0.99	0.06	0.10	22.49	90.85
6	205	0.62	0.73	0.05	0.55	0.22	0.72	0.05	0.11	20.32	84.50
7	219	0.45	0.38	0.03	0.40	0.18	0.38	0.03	0.08	20.32	90.25
8	567	0.66	3.32	0.14	0.40	0.43	3.36	0.14	0.14	53.59	88.61
9	554	0.85	2.14	0.09	0.41	0.68	2.13	0.09	0.25	53.64	86.51
10	1795	1.10	2.36	0.06	0.75	0.59	2.37	0.06	0.25	166.85	90.10
11	1777	1.24	3.19	0.08	0.55	1.12	3.15	0.07	0.51	166.60	89.33
12	234	0.87	3.80	0.25	0.40	0.76	3.82	0.25	0.17	21.68	90.38
13	214	0.54	1.36	0.09	0.40	0.41	1.36	0.09	0.23	21.78	82.31
14	235	2.33	9.04	0.59	0.66	1.95	9.01	0.59	0.20	22.11	89.00
15	241	1.02	2.80	0.18	0.42	0.86	2.77	0.18	0.28	22.15	91.12
16	271	3.37	9.31	0.57	0.63	3.33	9.23	0.56	0.71	25.76	88.11
17	273	1.52	3.25	0.20	0.64	1.22	3.17	0.19	0.43	25.79	88.64

Any use of trade, firm, or product names is for descriptive purposes only and does not imply endorsement by the U.S. Government.

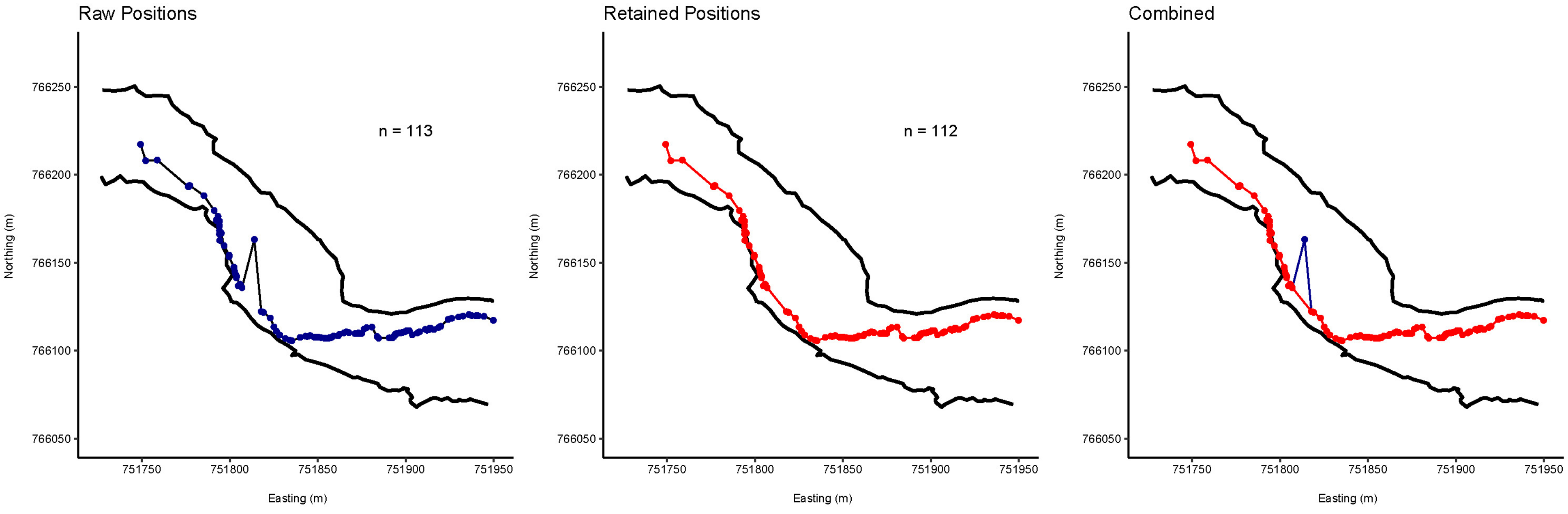

Fig. S4. Estimated sea lamprey positions and interpolated track before and after applying movement filters. Exemplary animal track made from (A) unfiltered and (B) movement speed filtered positions. (C) Filtered track overlay unfiltered track.

Journal of Experimental Biology • Supplementary information
